# Carbon-in-Silicate Nanohybrid Constructed by In Situ Confined Conversion of Organics in Rectorite for Complete Removal of Dye from Water

**DOI:** 10.3390/nano13192627

**Published:** 2023-09-23

**Authors:** Qingdong He, Jie Qi, Xiangyu Liu, Huan Zhang, Yiwen Wang, Wenbo Wang, Fang Guo

**Affiliations:** College of Chemistry and Chemical Engineering, Inner Mongolia University, Hohhot 010021, China; 18847744604@163.com (Q.H.); jieino@163.com (J.Q.); lxy13257244737@163.com (X.L.); hzhang1989@163.com (H.Z.); wangyiwen199501@163.com (Y.W.)

**Keywords:** rectorite, adsorbent, recycling, adsorption, wastewater

## Abstract

The complete removal of low concentration organic pollutants from wastewater to obtain clean water has always been a highly desired but challenging issue. In response to this, we proposed a new strategy to fabricate a carbon-in-silicate nanohybrid composite by recycling dye-loaded layered clay adsorbent and converting them to new heterogeneous carbon-in-silicate nanocomposite through an associated calcination-hydrothermal activation process. It has been confirmed that most of the dye molecules were present in waste rectorite adsorbent using an intercalation mode, which can be in situ converted to carbon in the confined interlayer spacing of rectorite. The further hydrothermal activation process may further improve the pore structure and increase surface active sites. As expected, the optimal composite shows extremely high removal rates of 99.6% and 99.5% for Methylene blue (MB) and Basic Red 14 (BR) at low concentrations (25 mg/L), respectively. In addition, the composite adsorbent also shows high removal capacity for single-component and two-component dyes in deionized water and actual water (i.e., Yellow River water, Yangtze River water, and seawater) with a removal rate higher than 99%. The adsorbent has good reusability, and the adsorption efficiency is still above 93% after five regeneration cycles. The waste clay adsorbent-derived composite adsorbent can be used as an inexpensive material for the decontamination of dyed wastewater.

## 1. Introduction

According to statistics, more than 100 tons of dye pollutants are discharged into the water environment every year [1]. The textile industry is the largest source of water pollution, followed by the paint, paper, leather, and printing industries [2]. These toxic dye wastewaters have caused great harm to human health, organisms, and ecosystems. How to economically and effectively remove dye pollutants in wastewater has become an urgent problem that needs to be solved [3,4,5,6]. The adsorption method is preferred as one of the most hopeful approaches for removing all kinds of pollutants owing to its simplicity, high efficiency, easy operation, reusability, and low cost [7,8,9].

In recent years, attempts have been made to remove dyes from wastewater with different adsorbent materials, such as activated carbon [10,11], polymer materials [12,13,14], modified clay [15], graphene [16], MOF [17], biochar [18], copper selenides [19], magnetic NiFe layered double hydroxide decorated diatomite [20], hybrid porous hexagonal boron nitride-based magnetic aerogel [21], and others. With the increasing demand for high water purification levels, the development of new adsorbents with a high dye removal rate, strong applicability in diverse water bodies, low cost, and wide range of sources has been pursued. Silicate clay minerals have been commonly recognized as low-cost and efficient adsorbents for the removal of pollutants due to their abundance, non-toxicity, stability, low cost, safety, and other advantages [22,23]. The clay minerals can be simply modified or compounded with other species to further elevate the adsorption capability. For example, the attapulgite/carbon composite shows an adsorption capacity (37.79 mg/g) and removal rate (95%) that is superior to raw attapulgite [24]. The magnetic magnesium-rich silicate adsorbent derived from natural attapulgite clay shows an adsorption capacity of 116.1 mg/g and 169.5 mg/g for methylene blue and cationic yellow dyes, respectively, and its performance is better than that of raw attapulgite clay [25]. The native advantages of clay minerals make them promising candidate for the development of high-performance adsorption materials.

Rectorite (R) is a unique two-dimensional layered clay mineral, which is composed of mica-like layers and montmorillonite-like layers in a ratio of 1:1 [26]. Compared with other layered clay minerals, it not only has cation exchange, water absorption, and swelling properties similar to montmorillonite, but also has high temperature-resistant properties similar to mica [27]. Rectorite is used to adsorb organic dyes in wastewater through hydrogen bond interactions, electrostatic attraction and ion exchange due to its pores, cation exchange capacity, surface negative charges, and surface silanol groups [28]. Although a series of natural clay minerals (such as rectorite) are widely used in the adsorption of dyes, the spent adsorbents will become solid waste, and its improper disposal will cause secondary pollution to the environment and a huge waste of mineral resources. Therefore, the effective disposal of the dye-adsorbed clay waste is extremely important [29], and relevant research have received much more attentions. Wang et al. synthesized a mesoporous silicate/carbon composite adsorbent from attapulgite-based waste dye adsorbent, which has a high removal capacity for tetracycline, methylene blue, and crystal violet [30]. Tian et al. use attapulgite (APT)-based dye adsorbent as a raw material to synthesize ternary attapulgite/carbon/silver nanoparticles (APT/C/AgNPs), nanocomposite with satisfactory adsorption and catalytic properties [31]. Zhai et al. converted methylene blue-adsorbed attapulgite at different temperatures to obtain a photocatalytic material with excellent efficiency for the degradation of bisphenol A [32]. These research proved a sustainable approach to convert waste to useful new materials. From previous research, it was also found that the conversion behavior of dye and the performance of the resulting adsorbent are highly dependent on the original structure of clay minerals. It is necessary to intensively examine the structure and performance of the composite derived from clay minerals with interlayered structures.

As a continuation of our systematic research work, this paper focused on the conversion and activation of waste rectorite adsorbent for production of highly efficient carbon-in-silicate adsorbent. The conversion behavior of dye in the interlayered clay was studied, the synthesis parameters were optimized, the change of microstructures of rectorite in the composites were explored. The adsorption behavior of the composite towards dyes from one-component and two-component system in different water medium was explored.

## 2. Materials and Methods

### 2.1. Materials

Rectorite (abbreviated as R) was obtained from Jingmen city, Hubei Province, China. Dye-loaded rectorite (MB-R) powder was obtained by the following process: rectorite powder and 1000 mL of MB dye solution (concentration, 5 g/L) were mixed together under continuous stirring for 24 h at room temperature to attain saturated adsorption. The solid was separated from the solution by a centrifugation process, and then crushed into a powder with a particle size < 76 µm. Methylene blue (MB) was bought from Adamas-beta Co., Ltd. in Shanghai, China. Basic Red 14 (BR) was bought from Duly Chemical Reagent Co., Ltd. in Nanjing, China. Hydrochloric acid (HCl aqueous solution) was purchased from Beijing Innochem Chemical Reagent Co., Ltd., in Beijing, China. Yangtze River water was sourced from the Jingzhou city, China. Yellow River water is taken from the Ordos city, China. Seawater is taken from the Bohai Sea, China (located at Tianjin, China). The parameters of the three actual water bodies are shown in Table S1 (Supplementary Material).

### 2.2. Synthesis of Carbon-in-Silicate Nanohybrid Composites

The composites were prepared via the following two-step process (Figure S1 in Supplementary Material). The first step is the carbonization process of dye-loaded rectorite. First, the MB-loaded rectorite powder (1 g) was placed in a tube furnace, and then calcined at 600 °C for 3 h under a nitrogen atmosphere (heating rate: 5 °C/min). The resultant black solid powder was marked as R/C. The second step is the activation of the R/C composite. The R/C powder (1 g) was dispersed into 20 mL of hydrochloric acid solution with different concentrations (0.5, 1, 2, 3, and 4 mol/L, respectively). The mixture was magnetically stirred for 30 min to obtain a uniform dispersion. Subsequently, the dispersion was transferred into hydrothermal reaction tank with Teflon liner (100 mL volume), then sealed and placed in an oven at 160 °C for different time intervals (2 h, 3 h, 4 h, 6 h, 8 h, and 12 h, respectively). After the hydrothermal process was completed, the reaction tank was naturally cooled to ambient temperature. The product was separated from the reaction mixture *via* a centrifugation process, washed several times with deionized water, dried, pulverized, and screened to obtain the final product. The product was labeled as R/C-xHAyh (x is the amount-of-substance concentration of HCl solution, mol/L; y is the hydrothermal reaction time, h).

### 2.3. Adsorption Experiments

#### 2.3.1. Adsorption Performance Test

The effects of pH, kinetics, and isotherm on adsorption experiments are detailed in the Supplementary Material.

#### 2.3.2. Test for Adsorption Efficiency of R/C-2HA4h Composite

Different doses of the composite adsorbents (0.5, 1.0, 1.5, 2.0, and 3.0 g/L, respectively) were added to the aqueous solution of MB or BR dye (concentration: 25 mg/L). After the adsorption process reaches equilibrium, the UV-Visible spectrum of the dye solution was scanned, the digital photograph of the solution was taken, and the removal rate of dyes after adsorption by the adsorbent was calculated. The adsorption capacity of the composite adsorbent (dose: 0.5 g/L) towards MB or BR dyes in different water bodies (i.e., Deionized water, Yangtze River water, Yellow River water, and Seawater) (dye concentration: 200 mg/L) was evaluated according to a similar procedure. The parameters of the three actual water bodies are shown in Table S2 (Supplementary Material). The co-adsorption properties of the composite towards the mixed dye solutions of MB and BR in different water medium was evaluated. Different doses of the composites (0.5 g/L, 1.0 g/L, 1.5 g/L, 2.0 g/L, 3.0 g/L, 4.0 g/L, and 5.0 g/L, respectively) were added to 20 mL of mixed dye solution (25 mg/L for MB and BR). After adsorption, the removal rate of dye by the adsorbent was tested and calculated, the UV-Visible spectrum of the solution was scanned, and the digital photograph of the solution was taken.

### 2.4. Characterizations

The instruments and methods used to characterize the composite adsorbents are shown in the Supplementary Material.

## 3. Results and Discussion

### 3.1. Structure and Morphology of Composites

**Figure 1:**
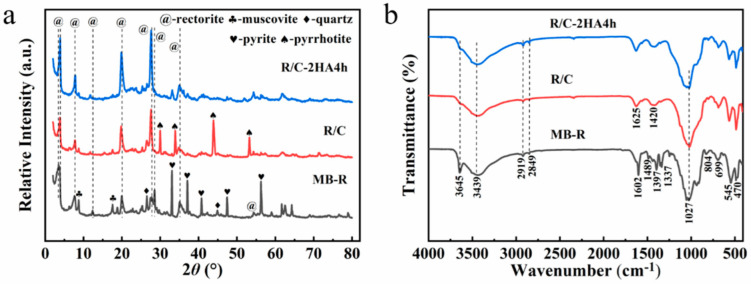
(**a**) XRD patterns of MB-R, R/C and R/C-2HA4h; (**b**) FTIR spectra of MB-R, R/C and R/C-2HA4h.

The XRD patterns of MB-loaded rectorite and R/C and R/C-2HA4h adsorbents are shown in Figure 1. The characteristic reflections of rectorite (2*θ* values: 3.48°, 7.66°, 19.94°, 27.40°, and 35.06°) [27] (JCPDS Card No. 29-1495), muscovite (2*θ* values: 8.74° and 17.50°) [30], quartz (2*θ* values: 26.46° and 44.84°) [25], and pyrite (2*θ* values: 33.02°, 37.04°, 40.74°, 47.40°, and 56.28°) (JCPDS No. 71-0053) [33] appeared in the XRD patterns of MB-R, which indicate that small amounts of associated minerals coexist with the rectorite mineral. After MB-R was calcined at 600 °C under nitrogen atmosphere, the diffraction peak of rectorite at 2*θ* = 3.48° (0 0 1 plane) shifts to larger angle, which confirms that the layer spacing decreases, and the MB in the interlayer spacing of rectorite was converted to carbon [29,34]. Figure 1a and Figure S2 (Supplementary Material) exhibit the XRD patterns of R/C after activation with different concentrations of acid solution and different hydrothermal activation times. When the concentration of acid solution and hydrothermal activation time gradually increase, the intensity of the diffraction peaks of (0 0 1) and (0 0 2) crystal planes decrease significantly, which is due to the partial removal of metal ions (such as Mg^2+^, Al^3+^) on the rectorite sheet during acid activation [35] and the formation of the amorphous region. When the concentration of acid solution is 4 mol/L, the reflection peaks of the (0 0 1) and (0 0 2) crystal planes disappear, indicating the acid activation process can remove some metal ions from the rectorite layer to create active adsorption sites that are beneficial to the adsorption of dyes.

Figure 1b shows the FTIR spectra of MB-R, R/C, and R/C-2HA4h. In the FTIR spectrum of MB-R, the bands at 3645 cm^−1^, 3439 cm^−1^, and 1023 cm^−1^ are the stretching vibration of Al-OH, the stretching vibration of O-H, and the stretching vibration of Si-O-Si, respectively [27]. The bands at 1336, 1395, 1489, and 1602 cm^−1^ are assigned to the antisymmetric and symmetric deformation bands of CH_3_, aromatic C-N, and aromatic C-C in MB, respectively [30,36,37]. The bands at 2919 cm^−1^ and 2849 cm^−1^ are the stretching vibration bands of C-H in -CH_3_ and -CH_2_, respectively [38]. After calcination treatment, the absorption bands of MB disappear, but the stretching vibration band of C-H in -CH_3_ and -CH_2_ still appears at 2917 cm^−1^ and 2849 cm^−1^, proving that the MB in the layer spacing of rectorite was converted to carbon after calcination. The Al-OH adsorption band at 3641 cm^−1^ almost disappears after calcination, which is due to the dehydroxylation reaction in the rectorite layer during the calcination process [27].

Figure S2c,d (Supplementary Material) shows the changes of functional groups on the adsorbent after R/C was activated with different concentrations of acid solution (fixed activation time: 8 h) and hydrothermal time (fixed acid concentration: 2 mol/L). With the gradual increase of acid concentration and the extension of activation time, the absorptive bands of Al-OH at 3641 cm^−1^ gradually disappeared, due to the dehydroxylation reaction of the rectorite layer [27], and the surface of rectorite was slightly etched. The stretching vibration bands of C-H in R/C at 2917 cm^−1^ and 2849 cm^−1^ almost disappeared after treatment with 4 mol/L of acid solution for 8 h, indicating that the high concentration of acid solution makes the carbon fall off by breaking the carbon/silicate interface.

**Figure 2:**
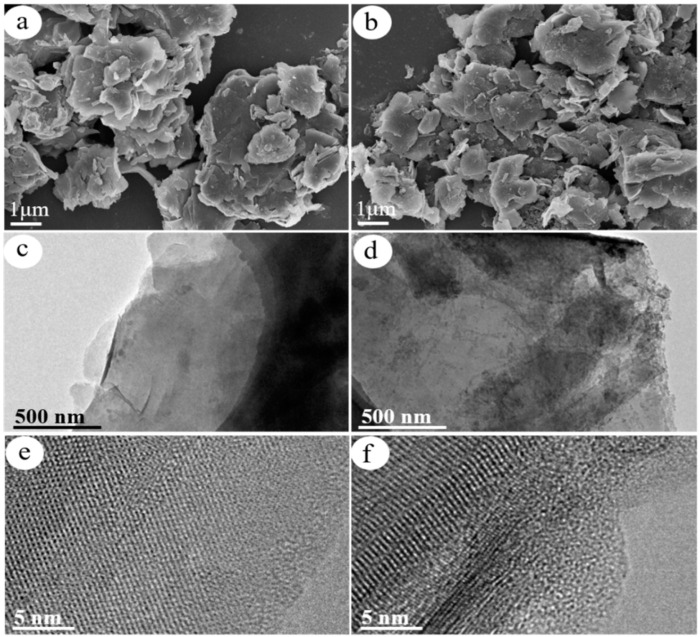
SEM images of (**a**) R/C and (**b**) R/C-2HA4h; and TEM images of (**c**,**e**) R/C and (**d**,**f**) R/C-2HA4h at different magnifications.

Figure 2a,b displays the SEM images of R/C and R/C-2HA4h, respectively. Rectorite maintains its complete lamellar structure after calcination process. However, its layer structure is slightly damaged after acid activation process, and some fragments were seen on it. Figure 2c,e and Figure 2d,f display the TEM images of R/C and R/C-2HA4h, respectively. The R/C composite still shows complete lamellar structure with uniform carbon layer (Figure 2c). After acid activation, the layer structure of rectorite is slightly damaged and the complete carbon layer is observed (Figure 2d). This is because the acid activation partially leached the metal ions (i.e., Mg^2+^, Al^3+^) on the surface of rectorite layer, and the layer structure is slightly changed. The amorphous region caused by acid etching are observed in the high magnification TEM picture of R/C-2HA4h (Figure 2f), which is favorable to increase the adsorption sites on the surface of rectorite. The EDS elemental analysis of R/C and R/C-2HA4h (Figure S3 in Supplementary Material) also proves the change of metal ions on the surface of rectorite layer. Before and after acid activation, the Mg and Al content on the surface of R/C decreased by 7.97% and 7.30%, respectively.

### 3.2. Raman Spectra Analysis

**Figure 3:**
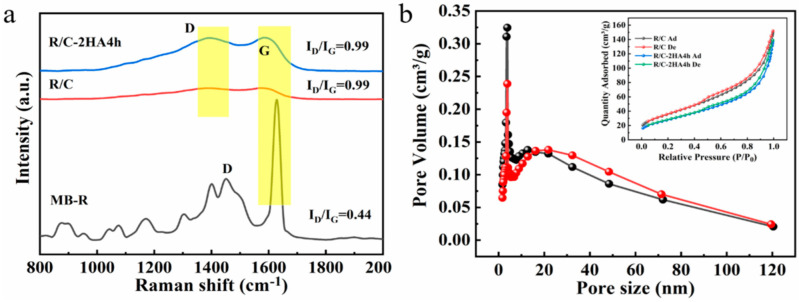
(**a**) Raman spectra of MB-R, R/C, and R/C-2HA4h; (**b**) the pore size distribution curves of R/C (black) and R/C-2HA4h (red) and N_2_ adsorption–desorption isotherms of R/C and R/C-2HA4h (Inset).

Raman spectroscopy is a commonly used method to study the structural characteristics of graphitic carbonaceous materials. Raman spectra of the MB-R, RE/C, and R/C-2HA4h composites were analyzed to reveal the presence of carbon in the materials. As shown in Figure 3a, the peaks at 1628, 1573, and 1584 cm^−1^ are identified as Raman shifts of G bands in MB-R, R/C, and R/C-2HA4h, respectively, which belong to the characteristic peak of graphitized carbon (sp^2^-C) [39]. The peaks at 1451, 1395, and 1392 cm^−1^ are identified as the Raman shifts of the D bands in MB-R, R/C, and R/C-2HA4h, respectively, which are ascribed to the absence of ordered carbon atoms or defective carbon atoms (sp^3^-C) [39]. The intensity ratio of D-band to G-band (I_D_/I_G_) can generally be used as an index to evaluate the order degree of carbon structure. The I_D_/I_G_ values of MB-R, R/C, and R/C-2HA4h are 0.44, 0.99, and 0.99, respectively. There are many peaks in the Raman spectrum of MB-R, which is due to the existence of MB in the MB-R, which is dominated by graphitized carbon. When MB-R was converted to R/C, the I_D_/I_G_ value was close to 1, and there were no other peaks in the Raman spectrum, accounting for the dye in MB-R being completely transformed into carbon. After the acid activation, the I_D_/I_G_ value of R/C-2HA4h is still the same as that of R/C, but the respective peak intensities have increased, indicating that the moderate acid activation process does not change the state of the carbon species. These results indicate that the carbon species in R/C and R/C-2HA4h are graphitized carbon and amorphous carbon [40].

### 3.3. BET Pore Structure Analysis

According to the IUPIC classification guideline, the R/C-2HA4h and R/C composites show type IIB isotherms with H_3_ hysteresis ring (Figure 3b) [41,42]. At the relative pressure *P*/*P*_0_ < 0.4, the adsorption–desorption curves almost coincided. When *P/P*_0_ > 0.4, the capillary condensation phenomenon caused by multilayer adsorption of nitrogen can be seen, indicating mesopores, micropores, and/or macropores are present in the R/C and R/C-2HA4h composites [43]. The pore-diameter distribution curves of the composites are shown in Figure 3b. The peak of R/C and R/C-2HA4h is centered at 3.8 nm, revealing that the composite is a mesoporous material. The pore structure parameters of R/C and R/C-2HA4h were listed in Table S2 (Supplementary Material). The average pore size of R/C-2HA4h is higher than R/C, which is beneficial for R/C-2HA4h to adsorb BR and MB.

### 3.4. Influence of Adsorption Conditions

#### 3.4.1. Influence of Synthesis Parameters

**Figure 4:**
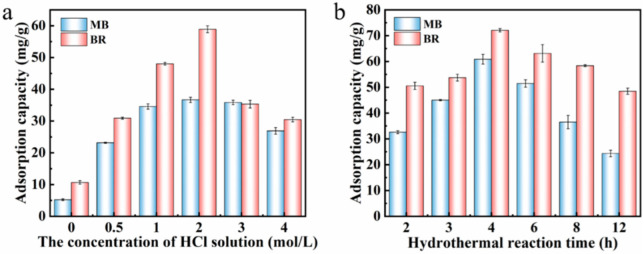
The effect of acid concentration (**a**) and activation time (**b**) on the adsorption amount of the as-prepared composites towards BR and MB dyes, respectively.

The effects of the concentration of acid solution and the activation time on the adsorption capacity were investigated (see Figure 4). When the concentration of acid solution was 2 mol/L and the acid activation time was 4 h, the adsorption amount of the adsorbent to dye reached the best values (60.87 mg/g for MB; and 72.10 mg/g for BR). In Figure 4a, the adsorption amount of the acid-activated adsorbent for dyes is better than that without acid activation. Taking the composite adsorbents prepared under the optimal reaction conditions as the optimal adsorbent, the effect of pH, adsorption kinetics, adsorption isotherm and other adsorption behaviors of the adsorbent were investigated.

#### 3.4.2. Effect of pH Values

**Figure 5:**
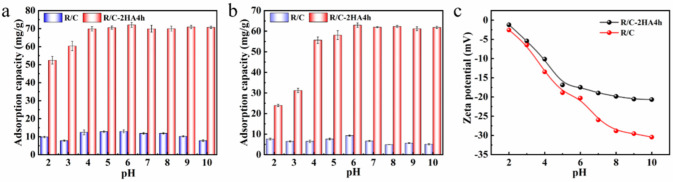
The adsorption capacity of the R/C and R/C-2HA4h composites (**a**) BR and (**b**) MB at variable pH; (**c**) Zeta potentials of the composite at variable pH.

The initial pH of the solution can affect the properties of dyes and the adsorbent, thus affect the adsorption behavior, especially for cationic dyes. As shown in Figure 5a,b, under each pH condition, the adsorption amount of R/C-2HA4h composite for dyes was much higher than that of R/C. In the test pH range (pH 2~10), the adsorption amount of R/C-2HA4h composite to BR is almost unaffected by the pH value, but it is slightly lower at pH 2~3, showing a very good pH immutability (Figure 5a). However, R/C-2HA4h composite is slightly pH-dependent for the adsorption of MB (Figure 5b). The adsorption capacity was relatively lower at pH 2~3, which increased rapidly with pH increasing from 2 to 4, and keeps almost constant at pH 4~10. The charge on R/C-2HA4h is negative (Figure 5c), which is conducive to the adsorption of positively charged cationic dyes. When pH is 2, because there are free H^+^ ions in the solution, some groups on the R/C-2HA4h change to Si-OH_2_^+^, which weaken the acting force between MB and the adsorbent, resulting in a slightly lower adsorption capacity towards MB [30]. When the pH value gradually increases to 6, the formed Si-OH_2_^+^ also slowly change to Si-O^−^ groups, and more negative charges are on the adsorbent surface [44], so it is easy to adsorb positively charged MB. The removal rate (r) of the adsorbents was compared with other adsorbents, as shown in Table 1. The r values of MB and BR of the R/C-2HA4h adsorbent were better than those of other adsorbents, which indicate that the adsorbent has better adsorption removal capability towards dyes.

#### 3.4.3. Adsorption Kinetics and Isotherms

**Figure 6:**
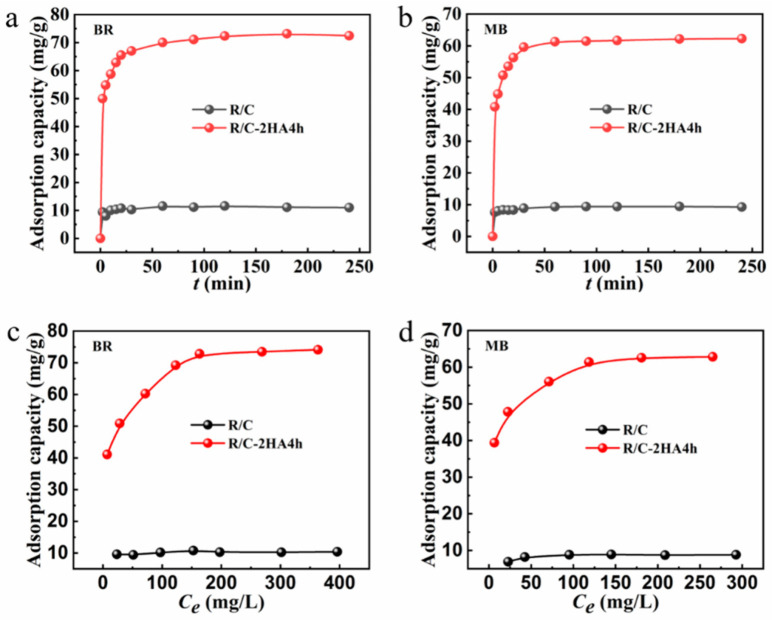
The change of adsorption amount of dye with altering adsorption time (**a**,**b**); The relationship curves of adsorption amount of BR and MB against the equilibrium concentration (*C*_e_, mg/L) (**c**,**d**).

Figure 6a,b shows the time-dependent adsorption behavior of BR and MB by R/C-2HA4h and R/C composites. The variation in the adsorption rate is strongly correlated with the solid–liquid concentration gradient between the dye solution and the adsorbent. In the initial stage, due to the large concentration gradient, more adsorbates will diffuse onto the surface of the adsorbent, and at the same time, the adsorbent has more adsorption sites to trap dye molecules. When the time gradually increased, the adsorption amount also increased rapidly. With the gradual progress of the adsorption, the concentration gradient between solid and liquid becomes smaller, leaching to a decrease of driving force for dye adsorption. Adsorption reaches equilibrium when all the adsorption sites were saturated [30]. The adsorption experiments of BR and MB by R/C-2HA4h adsorbent reached equilibrium within 30 min. Although R/C has poor adsorption capacity for BR and MB, the adsorption process reaches equilibrium rapidly (within 5 min). This may be because the fewer active adsorption sites on the surface of R/C can be rapidly occupied by dye molecules. When it is full, the adsorption reaches equilibrium immediately. As for adsorption kinetics, the experimental data were fitted using pseudo-first-order and pseudo-second-order kinetic models (Figure S4 in Supplementary Material) [57]. The fitting results with the pseudo-second-order model obtains a perfect linear relationship (*R*^2^ > 0.99), and the maximum adsorption amount calculated by the fitting is in agreement with the experimental value (Table S2 in Supplementary Material), which demonstrates that the kinetic adsorption behavior is consistent with the pseudo-second-order kinetic model, and chemisorption mainly contributes to the adsorption.

Figure 6c,d shows the variation curve of the adsorption capacity of dyes by the adsorbent with the equilibrium concentration of BR and MB after adsorption. In the initial stage, when the concentration of dye gradually increases, the adsorption amount also increases quickly. This is mainly because that the concentration gradient between the adsorbent and the adsorbate gradually increase, and the dye molecules are more easily diffused onto the surface of the adsorbent [30]. The adsorption of BR onto R/C-2HA4h adsorbent reached equilibrium (adsorption capacity: 72.83 mg/g) at the initial BR concentration of 200 mg/L, and the adsorption of MB onto R/C-2HA4h reached equilibrium (adsorption capacity: 61.39 mg/g) at the initial MB concentration of 150 mg/g. Beyond this concentration, the available adsorption sites are almost saturated, the adsorption amount no longer increases with increasing dye concentration [58]. For adsorption isotherm, the experimental data were fitted using Langmuir isotherm model and Freundlich isotherm model (Figure S5 in Supplementary Material) [59]. The adsorption amounts calculated by Langmuir isotherm model were in good agreement with the experimental values (Table S3 in Supplementary Material), and the *R*^2^ of the Langmuir model are all above 0.99. The results show that the adsorption behavior is consistent with the Langmuir isotherm model, in which the adsorption process belongs to the monolayer adsorption.

#### 3.4.4. Removal Efficiency

**Figure 7:**
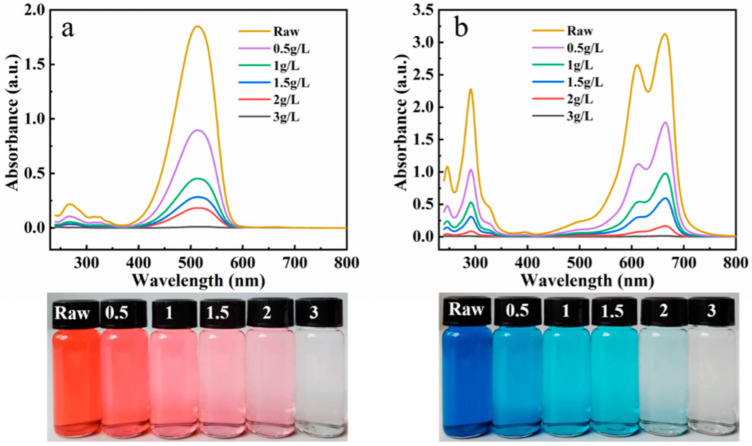
UV-Visible spectra and digital photos of the BR (**a**) and MB (**b**) solution before and after adsorption with different dose of R/C-2HA4h adsorbents.

The adsorption removal rate of MB and BR by the composite adsorbent was studied at the initial dye concentration of 25 mg/L and the variable adsorbent dosages (0.5, 1, 1.5, 2, and 3 g/L, respectively). After adsorption, the UV-Visible spectrum and digital photos of the solution were obtained (Figure 7a,b). The absorbance of the sample corresponding to the maximum absorption wavelength decreased with the increase in the amount of adsorbent, and the color of the solution became lighter. It is suggested that the removal rate can be improved by increasing the adsorbent dosage. When the adsorbent dosage was 3 g/L, both solutions (initial concentration: 25 mg/L) became colorless after adsorption (the removal rate of MB: 99.6%; the removal rate of BR: 99.5%), and the absorbance of the solution in the UV-Visible spectrum is almost zero. The adsorbent has a high removal rate for low-concentration dyes in solution, and has broad application prospects in the complete removal of dye effluent.

#### 3.4.5. Single/Co-Adsorption in Real Water and Deionized Water

**Figure 8:**
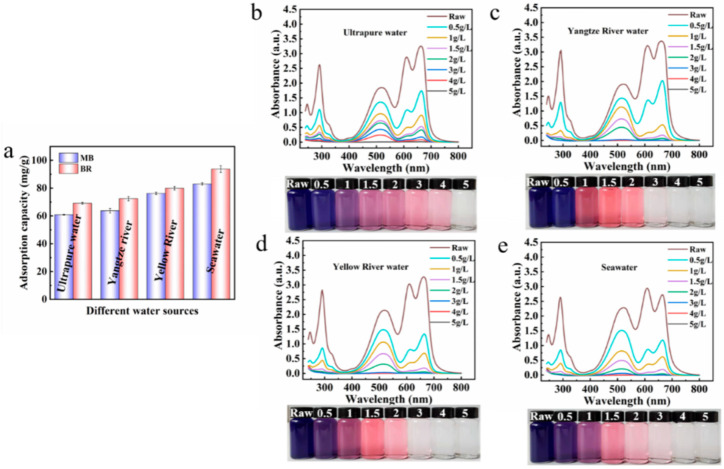
(**a**) The adsorption capacity of adsorbent for dyes in different water mediums (MB, 200 mg/L; BR, 200 mg/L; the dosage of adsorbent: 0.5 g/L). UV-Visible spectra of MB and BR mixed solution after adsorption with different dosages of R/C-2HA4h in deionized water (**b**), Yangtze River water (**c**), Yellow River water (**d**), and Seawater (**e**) (MB, 25 mg/L; BR, 25 mg/L). The illustrations are digital photos taken before and after adsorption of the binary mixed dye solution.

Due to the complexity of the actual water environment, the adsorption performance of the adsorbent in the actual water body is very important. The adsorption performance of the adsorbent towards MB and BR in three actual water samples (Yangtze River water, Yellow River water, and Seawater) were investigated (Figure 8). Encouragingly, the adsorption performance of the composite adsorbent for both dyes in the actual water body is better, which may be due to the adsorbent having a stronger interaction with the dye molecules [60]. In addition, the adsorption property of the R/C-2HA4h adsorbent for MB and BR dyes in mixed solutions (MB or BR concentrations: 25 mg/L) were compared using three actual water samples and deionized water as solvent. As shown in Figure 8b–e, after adsorption with R/C-2HA4h adsorbent, when the amount of adsorbent increased gradually, the absorbance of the mixed dye solution decreased, and the color of the solution gradually changed light. In the three actual waters, when the dosage of the adsorbent was 3 g/L, the dye solution becomes colorless, and no absorbance peak was observed in the UV-Visible spectrum (the absorbance is almost 0) (Figure 8c–e). At this point, above 99.9% of the dye in the binary mixed solution was removed. In the deionized water (Figure 8b), the dye solution becomes colorless when the amount of the adsorbent was 5 g/L, and the dye in the binary mixed solution is completely removed at this time. For the adsorption of dyes in actual water, the dyes can be removed with a less amount of adsorbent. Therefore, the single/co-adsorption property of the composite material in real water and deionized water is better, which suggests its potential to be used for the removal and purification of multiple dyes in wastewater.

#### 3.4.6. Reusability and Cost Analysis

After MB and BR dye solution (concentration: 400 mg/L) were adsorbed by the R/C-2HA4h adsorbent (1 g), the spent adsorbent can be regenerated via a calcination process at 400 °C followed by a washing process (Figure S1 in Supplementary Material). The adsorption-regeneration process is conducted five times, and the adsorption efficiency of each regenerated adsorbent was tested to evaluate the reusability of the adsorbent. As shown in Figure S7 (Supplementary Material), after five regeneration–adsorption cycles, the adsorption efficiency of the adsorbent towards dyes can still reach more than 93%, which confirms that the adsorbent has good reusability.

Through a simple cost–benefit analysis, the practical application potential of the composite adsorbent was discussed. The cost analysis results of the composite adsorbent are shown in Table S5 (Supplementary Material). Since the main raw material for preparing the composite adsorbents is dye–clay waste, and clay has huge reserves in nature, the cost advantage of the adsorbent is obvious. The price of R/C-2HA4h composite adsorbent is about $475/ton, which is far lower than the current average price of commercial activated carbon (≥$1200/ton). In addition, the composite adsorbent has more advantages in the removal of dyes in practical wastewater. Therefore, the composite adsorbent with good performance and economic benefits has a great potential for efficient purification of complex dye wastewater.

#### 3.4.7. Adsorption Mechanism

**Figure 9:**
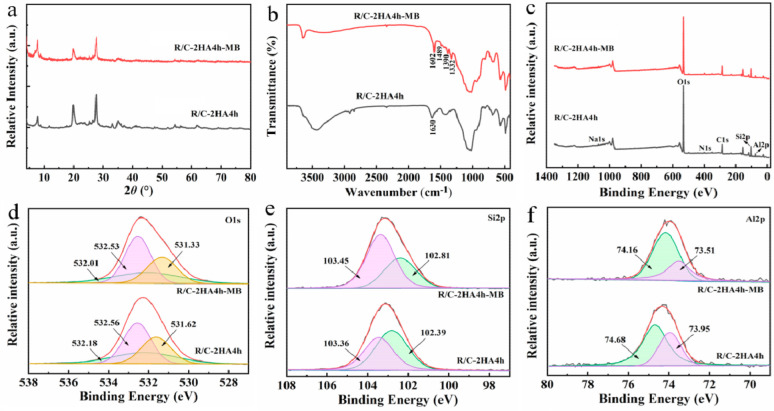
(**a**) XRD patterns of R/C-2HA4h and R/C-2HA4h-MB; (**b**) FTIR spectra of R/C-2HA4h and R/C-2HA4h-MB; Survey scanning XPS spectra of R/C-2HA4h and R/C-2HA4h-MB (**c**); and fine-scanning XPS spectra of O 1s (**d**), Si 2p (**e**), and Al 2p (**f**) of R/C-2HA4h and R/C-2HA4h-MB.

**Figure 10:**
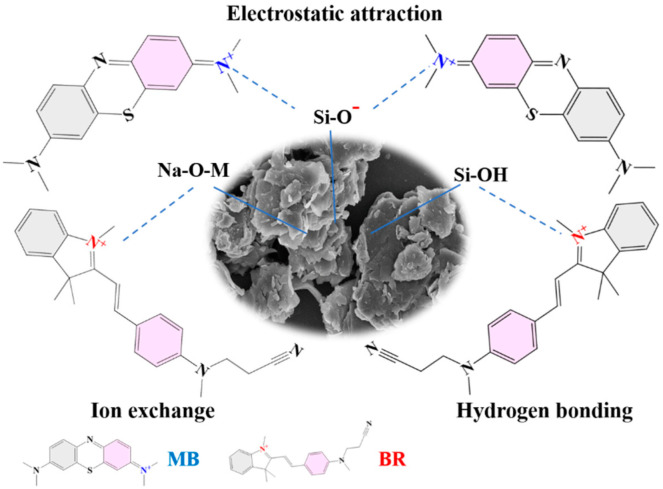
Interaction mechanism diagram of R/C-2HA4h adsorbent with MB and BR.

The adsorption mechanism was studied by analyzing the dye-adsorbed adsorbent with XRD, FTIR spectra, BET, and XPS spectra. The diffraction peak of MB crystal is not seen in the XRD pattern of R/C-2HA4h-MB (Figure 9a), which shows that the MB dye is adsorbed on the adsorbent in a molecular form. It can be seen from the FTIR spectrum of R/C-2HA4h-MB (Figure 9b), after adsorption of MB, the new absorption bands appear at around 1602, 1489, 1390, and 1332 cm^−1^ (the stretching vibration of aromatic C-C, the stretching vibration of aromatic C-N, and the antisymmetric and symmetric deformation bands of CH_3_ in MB, respectively) [30,36,37]. At 1630 cm^−1^ (H–O–H bending), the absorption band of the adsorbent weakened slightly and shifted toward lower wavenumber region, demonstrating that there are hydrogen-bonding interactions between R/C-2HA4h and dyes. As shown in Table S2 (Supplementary Material), when the dye was adsorbed onto the adsorbent, the BET specific surface area decreased, indicating that dye molecules entered the pores. This also shows that the pore plays an important role in the adsorption of dye. The full-scan XPS spectra showed that the composition elements of R/C-2HA4h adsorbent are Si, Al, O, C, N, and Na (Figure 9c). After adsorbing the dye, the signal peaks of these elements all moved towards the low binding energy region, which was caused by the interaction of MB dye molecules with surface groups of rectorite during the adsorption process. The O 1s peak of R/C-2HA4h is divided into Si-OH, Si-O-Al, and Si-O-Si, which are located at 531.62, 532.18, and 532.56 eV, respectively [61], and they move to 531.33, 532.01, and 532.53 eV, respectively, after adsorption (Figure 9d). The Si 2p peak of R/C-2HA4h are divided Si-O-Al and Si-O-Si, which are located at 102.39 eV and 103.36 eV, respectively. After adsorption of MB, these peaks shift to 102.81 eV and 103.45 eV, respectively (Figure 9e). At the same time, the Al 2p peak of R/C-2HA4h and R/C-2HA4h-MB can be divided into two peaks, located at 74.68 eV (Si-O-Al) and 73.95 eV (Si-O-Al-O) and 74.16 (Si-O-Al) and 73.51 eV (Si-O-Al-O) (Figure 9f), respectively [62]. After adsorption of MB, the binding energy of these cleavage peaks shifts, and the peak area changes. This change is visible as aerobic groups are involved in the adsorption process of MB on R/C-2HA4h. In general, the main adsorption mechanisms of the composite adsorbents to dyes are electrostatic attraction, ion exchange, and hydrogen bond interaction between adsorbent and dye [63]. As for the adsorption of the composite adsorbent towards cationic dyes, the electrostatic attraction, ion exchange, hydrogen bonding, and chemical interaction between Si-O− group and dye group mainly contribute to the adsorption process (Figure 10). Therefore, the adsorbent has a good ability and potential to remove dyes.

## 4. Conclusions

Dye-loaded layered clay adsorbent waste was converted into a new carbon-in-silicate nanohybrid composite with ultrahigh removal ability for dyes at low concentrations. The dye intercalated in the interlayer spacing of rectorite can be converted into carbon sheet within the silicate, and the associated acid activation process can further create defects to produce more adsorption sites, which enable the resultant composite to completely remove dye in wastewater. Therefore, the composite adsorbent has a very high removal rate of 99.6% and 99.5% for MB and BR dyes (initial concentration: 25 mg/L), respectively. The adsorption of dyes by the composite adsorbents conformed to the Langmuir isotherm model and the pseudo-second-order kinetic model, indicating that the adsorption process is a typical monolayer adsorption process, and chemisorption action is dominant. In addition, the composite adsorbent also shows high removal capacity for dyes in single-component and two-component solution system in deionized water and actual water (i.e., Yellow River water, Yangtze River water, and seawater) with a removal rate higher than 99%. The adsorbent has good reusability, and the adsorption efficiency is still above 93% after five regenerations. Moreover, the composite adsorbent is prepared from solid waste, so it has great potential for the purification of practical printing and dyeing wastewater.

## Figures and Tables

**Table 1:** The comparison of removal rates (r) of dyes using different adsorbents at the optimal pH.

Adsorbents	Dyes	Initial Concentration (mg/L)	r (%) (optimal pH)	Ref.
F-CNTs@MOF@Gel	MB	100	81 (pH 7.89)	[45]
MnFe_2_O_4_/rGO	MB	100	95 (pH 7)	[46]
Tailing Ash	MB	20	96.2 (pH 10)	[47]
PpAP/Starch/GO	MB	100	96.7 (pH 7)	[48]
CNS/(PAAc-MAC)	MB	50	98 (pH 7)	[49]
Algal Biochar	MB	100	97.5 (pH 7)	[50]
Carbonized Dacryodes edulis leaf	MB	100	93 (pH 4)	[51]
R/C-2HA4h	MB	25	99.6 (pH 6)	This work
Valorization of olive–pomace	BR	200	90 (pH 7)	[52]
CuO nanoparticles	BR	10	80 (pH 7)	[53]
MCM-48	BR	500	97 (pH 4.6)	[54]
O-CM-chitosan hydrogel	BR	400	85 (pH 7)	[55]
Orange peel biochar	BR	100	94 (pH 10)	[56]
R/C-2HA4h	BR	25	99.5 (pH 6)	This work

## Data Availability

The data presented in this study are available upon request from the corresponding author.

## Supplementary Materials

The following supporting information can be downloaded at: https://www.mdpi.com/article/10.3390/nano13192627/s1. Figure S1: Preparation diagram and regeneration cycle experiment diagram of R/C-2HA4h adsorbent; Figure S2: XRD patterns of R/C, and the composite adsorbents prepared at different concentration of acid solution (a) and acid activation time (b); FTIR spectra of R/C, and the composite adsorbents prepared at different concentration of acid solution (c) and activation time (d). Figure S3: The EDS image of (a) R/C and R/C-2HA4h(b). Figure S4: The plots of log(*q*_e_-*q*_t_) versus *t* for the adsorption of BR (a) and MB (c) on the composite adsorbents (fitting with pseudo-first-order model); and the plots of *t*/*q*_t_ versus *t* for the adsorption of BR (c) and MB (d) on the composite adsorbents (fitting with pseudo-second-order model). Figure S5: The plots of *C*_e_/*q*_e_ versus *C*_e_ for the adsorption of BR(a) and MB(c) on the composite adsorbents (fitting with Langmuir model); and the plots of log*q*_e_ versus log *C*_e_ for the adsorption of BR(b) and MB(d) on the composite adsorbents (fitting with Freundlich model). Figure S6: N_2_ adsorption–desorption isotherms of R/C-2HA4h and R/C-2HA4h-MB(a), the pore size distribution curves of R/C-2HA4h and R/C-2HA4h-MB (b). Figure S7: Recycling efficiency diagram of R/C-2HA4h. Table S1: pH and electrical conductivity of Yangtze River water, Yellow River water, Sea water. Table S2: Specific surface area and pore structure parameters for R/C, R/C-2HA4h, and R/C-2HA4h-MB. Table S3: Adsorption kinetic parameters calculated from the fitting results with pseudo-first-order and pseudo-second-order kinetic model for adsorption of BR and MB. Table S4: Adsorption isotherm parameters calculated from the fitting results of Langmuir and Freundlich model for adsorption of BR and MB. Table S5: Commercial cost—benefit analysis of composite adsorbents in dye wastewater purification in China. (see Refs. [64,65,66,67]).
