# Supplementary material for: Carbon-in-Silicate Nanohybrid Constructed by In Situ Confined Conversion of Organics in Rectorite for Complete Removal of Dye from Water
Source: Nanomaterials (Basel). 2023 Sep 23;13(19):2627. doi: 10.3390/nano13192627 (PMC10574537; doi:10.3390/nano13192627)
Supplement: Supplementary file 1 [file nanomaterials-13-02627-s001.zip › nanomaterials-2612848-supplementary.pdf]

# Carbon-in-Silicate Nanohybrid Constructed by in Situ Confined Conversion of Organics in Rectorite for Complete Removal of Dye from Water

**Qingdong He,† Jie Qi,† Xiangyu Liu, Huan Zhang, Yiwen Wang, Wenbo Wang,* Fang Guo***

College of Chemistry and Chemical Engineering, Inner Mongolia University, Hohhot, 010021, P.R. China; 18847744604@163.com (Q.H.); jieino@163.com (J.Q.); 1446378431@qq.com (X.L.); hzhang1989@163.com (H. Z.); wangyiwen199501@163.com (Y.W.); wangwenbo@imu.edu.cn (W.W.); guofang@imu.edu.cn (F. G.)

*Correspondence: wangwenbo@imu.edu.cn (W.W.); guofang@imu.edu.cn (F.G.)

†These authors contribute equally to the paper.

## Structure characterizations

A Nicolet IS 10 Infrared spectrometer (USA) was used to collect the FTIR spectra (wavenumber range: 4000-400 cm-1). The samples were prepared as KBr pellets for FTIR test. The ZEISS SUPRA55 Field Emission Scanning Electron Microscope (SEM, Germany) was used to observe the surface morphology of samples. The samples were fixed on copper stubs and coated with gold nanoparticles to increase the conductivity. The JEM-2100f high-resolution Transmission Electron Microscope (Japan) was used to take the TEM images. Before observation, the sample was ultrasonically dispersed in anhydrous ethanol and dropped onto a micro grid. XRD patterns were collected from 2° to 80° using an X'Pert PRO diffractometer (PANnalytical Co., Germany) equipped with a Cu-K$\alpha$ radiation source (40 kV, 40 mA) in a step interval of approximately 0.167° and a fixed time mode. The specific surface area was determined using an ASAP 2020 M instrument (Micromeritics Instrument Corporation, USA) by the Brunauer-Emmett-Teller (BET) method, while the pore volume and pore diameter was estimated by the Barrett-Joyner-Halenda (BJH) method at 77 K. The BET specific surface area (BET) was calculated by the BET equation; the total pore volumes ($V_{total}$) were obtained from the volume of liquid $N_2$ held at the relative pressure ($P/P_0$) of 0.95. the average pore diameter was calculated according to BET method. Before performing BET test, 0.1 g samples were dried to constant weight in a vacuum oven at 100 °C.

## Adsorption performance test

The solid adsorbents (0.0100 g) were added to 20 mL of the target dye solutions, and then the mixture was oscillated on a Shaking Chamber (THZ-98A, Yiheng, Shanghai) at 30 °C. After 4 h, the adsorbent was separated from solution, and the concentration of dyes in the filtrate was measured with an UV-Vis Spectrophotometer (UV1900i, Shimadzu). The adsorption amount of MB or BR on per unit mass of the adsorbent ($q_e$, mg/g) was calculated using the following equation (S1).

$$Q_e = [(C_0 - C_e) \times V]/m \quad (S1)$$

In this equation, $C_0$ denotes the initial concentration of MB solution (mg/L); $C_e$ denotes the concentration of MB solution at adsorption equilibrium state (mg/L); $V$ is the volume of solution used (L); and m is the mass of adsorbent used for adsorption (g), respectively.

The effects of adsorption parameters such as initial pH, contact time and initial dye concentration on the adsorption performance of the composite adsorbents towards MB or BR were studied. To evaluate the effect of external pH values, the pH of MB and BR solution is changed from 2 to 10 and the initial concentration of dye solution was fixed at 200 mg/L.

To evaluate the effect of initial concentration on adsorption, The initial concentration of adsorbents adsorption of MB changes from 25 to 300, The initial concentration of adsorbents adsorption of BR changes from 25 to 400. Langmuir and Freundlich isotherm models were used to fit the adsorption experimental data and study the adsorption isotherms. The linear form of Langmuir isotherm model is given by the following equation (S2) [1]:

$$\frac{C_e}{q_e} = \frac{1}{q_m k_L} + \frac{C_e}{q_m} \tag{S2}$$

$q_m$ and $k_L$ represent Langmuir maximum adsorption capacity and Langmuir constant related to adsorption heat of the adsorbent, respectively. $C_e$ is the concentration of the dye solution at equilibrium state (mg/L).

The linear form of Freundlich isotherm model [2] is given by the following equation (S3):

$$\log q_e = k_F + \frac{1}{n}\log C_e \tag{S3}$$

$k_F$ (L/g) and $n$ are Freundlich constants corresponding to adsorption capacity and adsorption strength, respectively.

The effect of contact time on the adsorption was studied in the time range of 2-240 min. In order to study the kinetic adsorption process of BR and MB dyes on the composite adsorbents, the pseudo-first-order and pseudo-second-order kinetic models were used to fit the experimental adsorption data and describe the dependence of adsorption capacity on adsorption time. The pseudo-second-order and pseudo-first-order adsorption kinetic models were expressed as follows [3,4].

Pseudo-first-order kinetic model:

$$\log(q_e - q_t) = \log q_e - k_1 t/2.303 \tag{S4}$$

Pseudo-second-order kinetic model:

$$\frac{t}{q_t} = 1/k_2 {q_e}^2 + 1/q_e t \tag{S5}$$

Where $q_t$ (mg/g) is the adsorption amount of MB and BR at time $t$; $q_e$ (mg/g) is the adsorption capacity of MB and BR at adsorption equilibrium (mg/g); $k_1$ is the rate constant of the pseudo-first-order kinetic model; $k_2$ is the rate constant of the pseudo-second-order kinetic model.

## Supplementary Figures

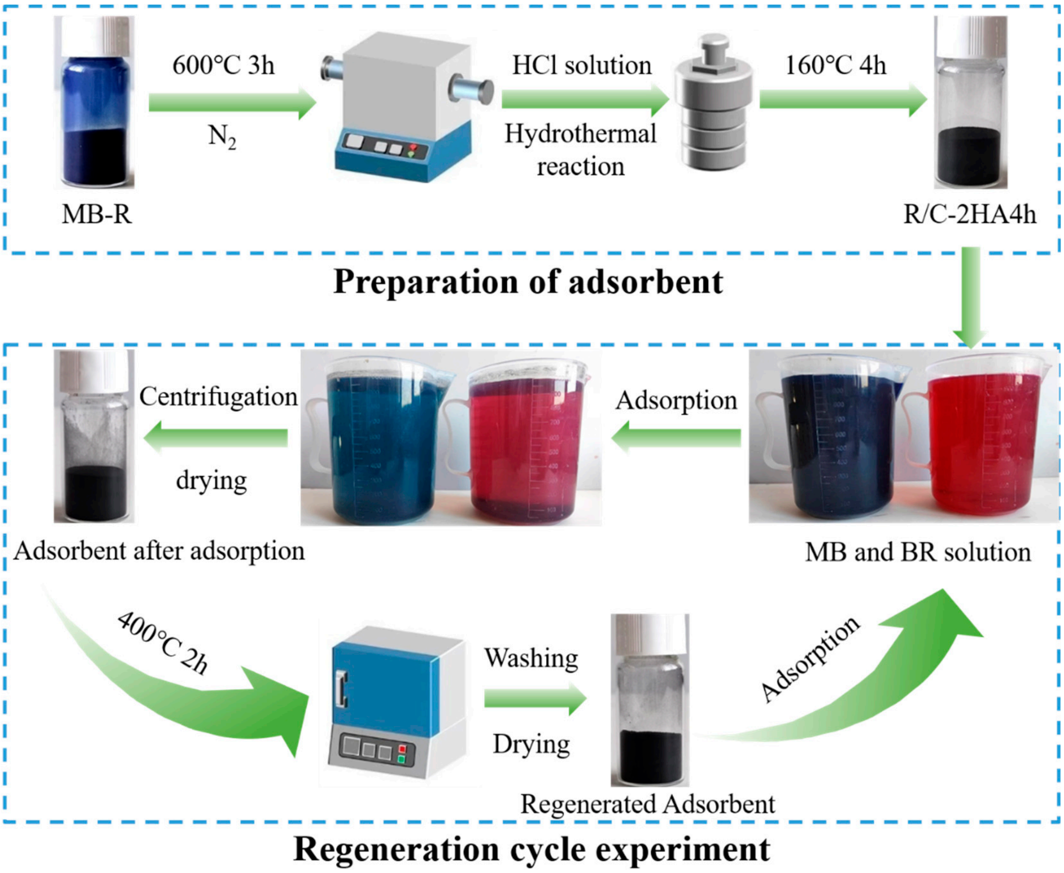


**Figure S1.** Preparation diagram and regeneration cycle experiment diagram of R/C-2HA4h adsorbent

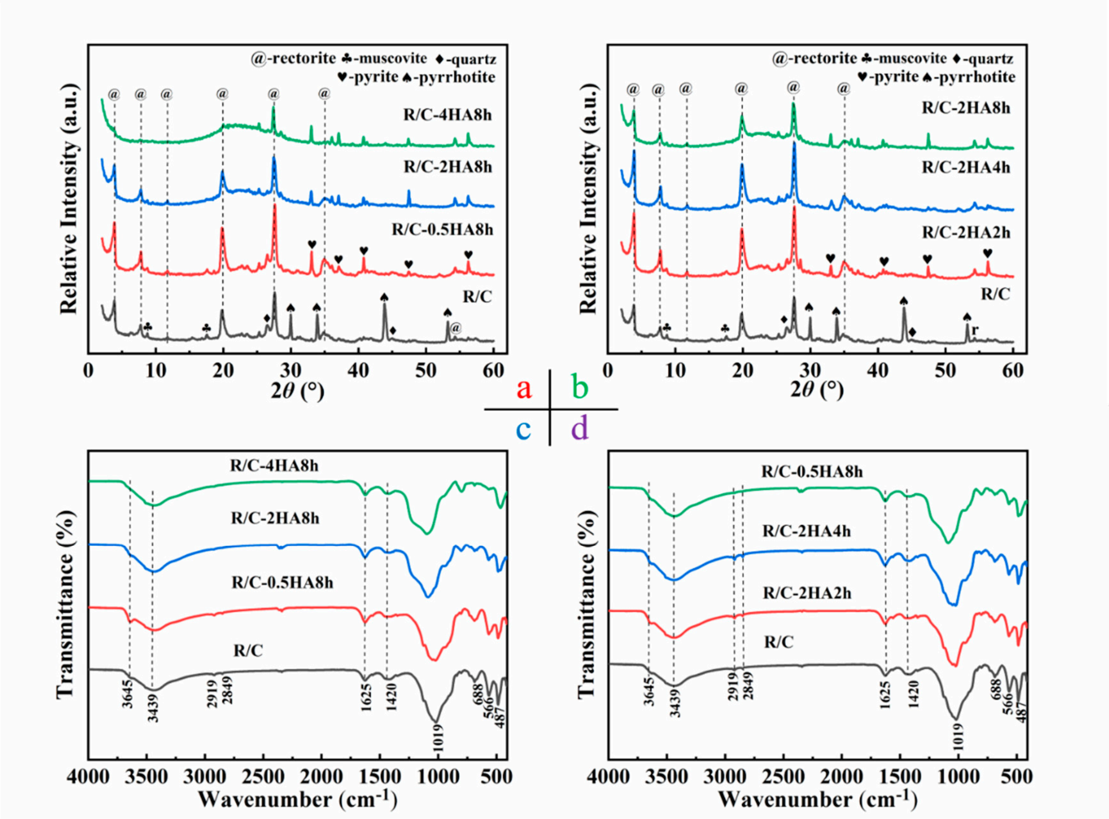


**Figure S2.** XRD patterns of R/C, and the composite adsorbents prepared at different concentration of hydrochloric acid solution (a) and hydrothermal treatment time (b). FTIR spectrum of R/C, and the composite adsorbents prepared at different concentration of hydrochloric acid solution (c) and hydrothermal treatment time (d).

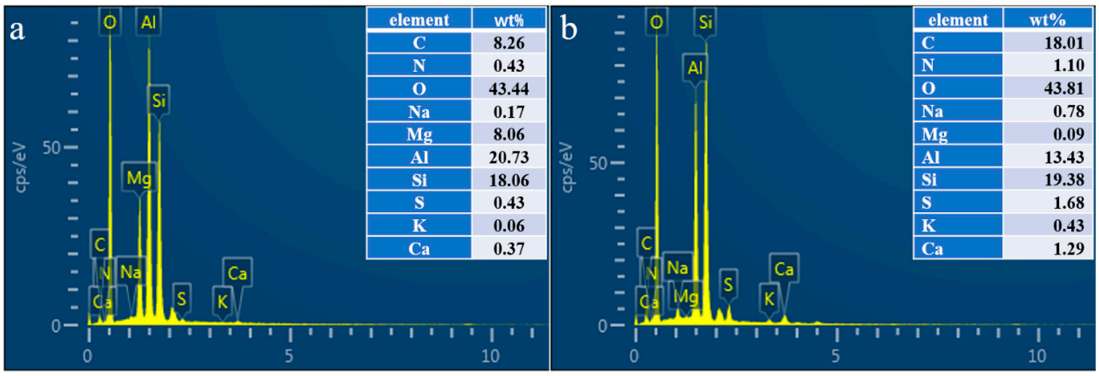


**Figure S3.** The EDS image of (a) R/C and R/C-2HA 4h(b)

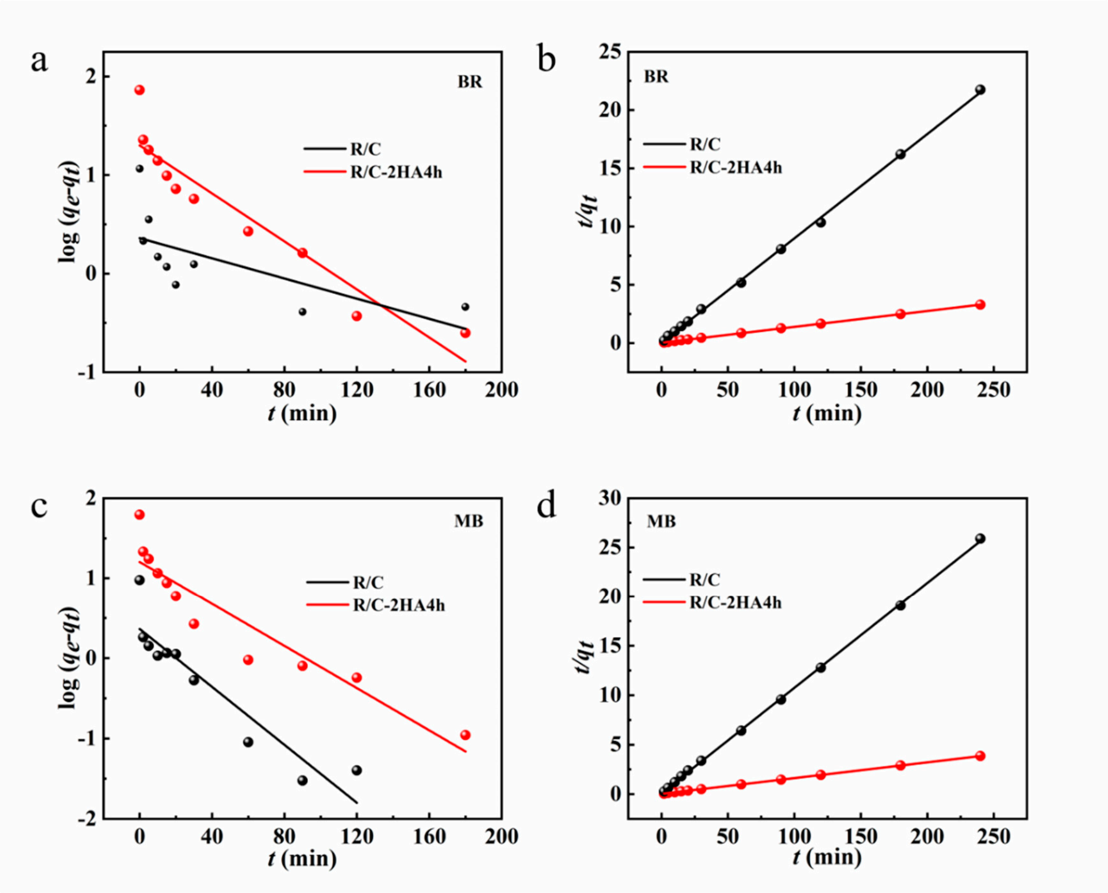


**Figure S4.** The plots of log($q_e$-$q_t$) versus $t$ for the adsorption of BR(a) and MB(c) on the composite adsorbents (fitting with pseudo-first-order model); The plots of $t/q_t$ versus $t$ for the adsorption of BR(c) and MB(d) on the composite adsorbents (fitting with pseudo-second-order model)

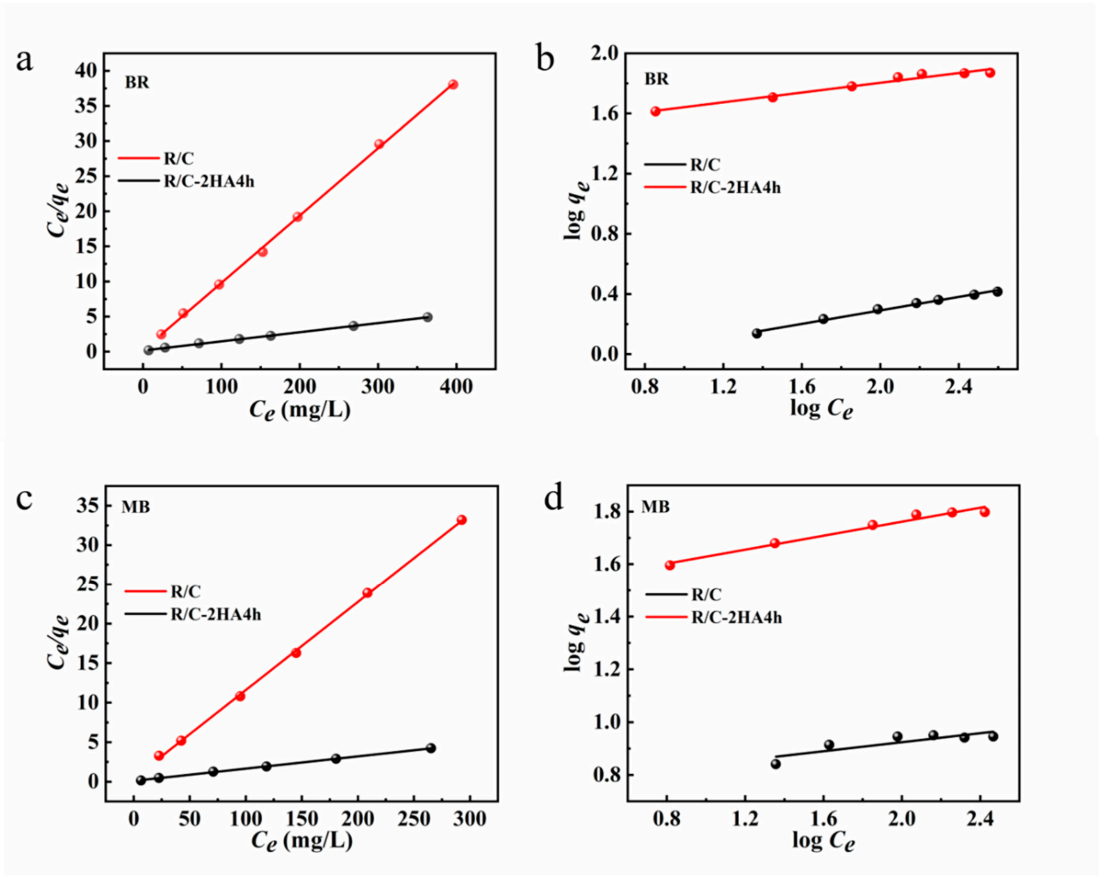


**Figure S5.** The plots of $C_e/q_e$ versus $C_e$ for the adsorption of BR(a) and MB(c) on the composite adsorbents (fitting with Langmuir model); and the plots of log $q_e$ versus log $C_e$ for the adsorption of BR(b) and MB(d) on the composite adsorbents (fitting with Freundlich model).

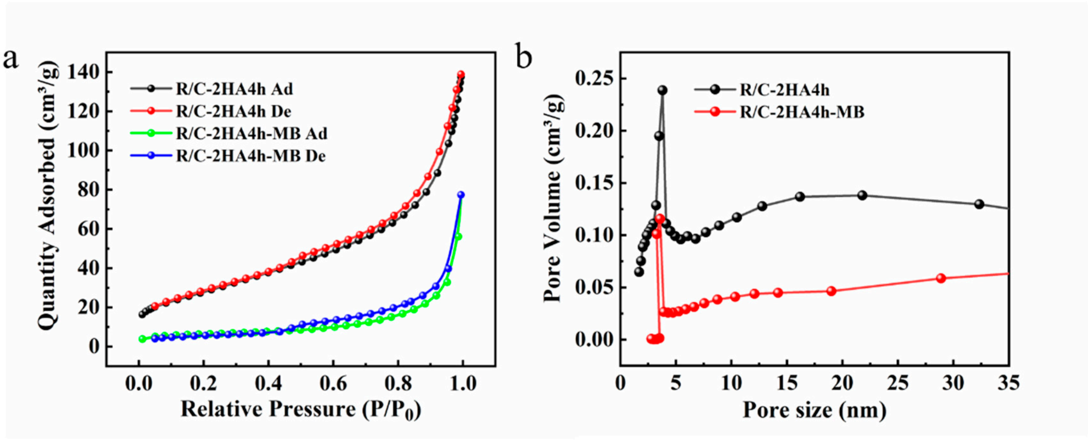


**Figure S6.** $N_2$ adsorption-desorption isotherms of R/C-2HA4h and R/C-2HA4h-MB (a), the pore size distribution curves of R/C-2HA4h and R/C-2HA4h-MB (b).

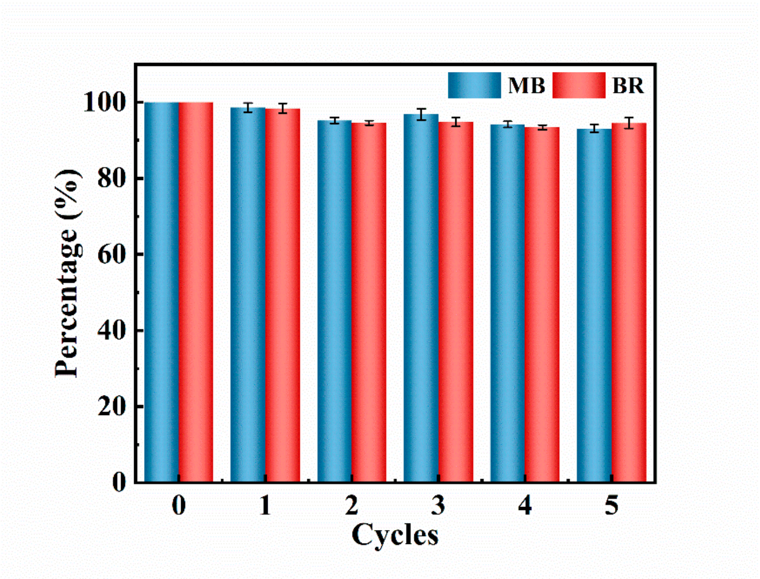


**Figure S7.** Recycling and regeneration efficiency diagram of R/C-2HA4h

## Supplementary Tables

**Table S1.** pH and conductivity of Yangtze River water, Yellow River water, Sea water

| Parameters | Yangtze River water | Yellow River water | sea water |
|---|---|---|---|
| pH | 7.63 | 8.13 | 7.57 |
| Conductivity | 342.9 μS/cm | 659.7 μS/cm | 31.29 mS/cm |

**Table S2**. Specific surface area and pore structure parameters for R/C, R/C-2HA4h and R/C-2HA4h-MB

| Samples | $S_{BET}$ ($m^2/g$) | Average pore size (nm) | Pore Volume ($cm^3/g$) |
|---|---|---|---|
| R/C | 129.57 | 7.26 | 0.24 |
| R/C-2HA4h | 100.91 | 8.07 | 0.20 |
| R/C-2HA4h-MB | 22.46 | 8.93 | 0.05 |

**Table S3.** Adsorption kinetic parameters calculated from the fitting results with pseudo-first-order and pseudo-second-order kinetic model for adsorption of BR and MB

| Adsorbate | Samples | Pseudo-first-order model | | | Pseudo-second-order model | | |
|---|---|---|---|---|---|---|---|
| | | $k_1$ ($min^{-1}$) | $q_{cal,1}$ (mg/g) | $R^2$ | $k_2$ ($min^{-1}$) | $q_{cal,2}$ (mg/g) | $R^2$ |
| BR | R/C | 0.01176 | 2.30 | 0.3771 | 0.187 | 11.17 | 0.9992 |
| | R/C-2HA4h | 0.02802 | 19.96 | 0.8853 | $6.157\times10^{-3}$ | 73.37 | 0.9999 |
| MB | R/C | 0.04150 | 2.31 | 0.8499 | 0.102 | 9.40 | 0.9997 |
| | R/C-2HA4h | 0.03030 | 15.96 | 0.8643 | $8.251\times10^{-3}$ | 62.77 | 0.9999 |

**Table S4.** Adsorption isotherm parameters calculated from the fitting results of Langmuir and Freundlich model for adsorption of BR and MB

| Adsorbate | Samples | Langmuir model | | | Freundlich model | | |
|---|---|---|---|---|---|---|---|
| | | $k_L$(L/mg) | $q_m$(mg/g) | $R^2$ | $k_F$ $(L/mg)^{1/n}$ | n | $R^2$ |
| BR | R/C | 0.467 | 10.43 | 0.9991 | 0.696 | 4.46 | 0.9894 |
| | R/C-2HA4h | 0.081 | 76.69 | 0.9987 | 30.184 | 6.17 | 0.9625 |
| MB | R/C | 0.257 | 8.96 | 0.9994 | 5.666 | 11.71 | 0.6654 |
| | R/C-2HA4h | 0.144 | 64.56 | 0.9994 | 31.331 | 7.53 | 0.9697 |

**Table S5.** Commercial cost - benefit analysis of composite adsorbents

| Types (e.g., raw materials, energy consumption, labor) | Unit Price | Usage amounts | Cost |
|---|---|---|---|
| Waste sorbents | $0/ton | 2 ton | $0 |
| Hydrochloric acid | $137.9/ton | 2.8 ton | $386.12 |
| Water | $0.29/ton | 20 ton | $5.8 |
| Nitrogen | $1.38/L | 40 | $55.2 |
| Electron energy consumption | $0.043/kWh | 500 | $21.5 |
| Labor cost | $2.5/ton | 1 | $2.5 |
| Total cost | | | $471.12/ton |

**References**


1. Langmuir, I. The Constitution and fundamental properties of solids and liquids. II. LIQUIDS.1. *Journal of the American Chemical Society* **1916**, *39*.
2. Freundlich, H. Over the adsorption in solution. *J. Phys. Chem.* **1906**, *57*, 1100-1107.
3. Ho, Y.S. A review of potentially low cost adsorbent for heavy metals. *Scientometrics* **2004**, *59*, 171-177.
4. Ho, Y.S.; Fellow, M.K. A Comparison of Chemisorption Kinetic Models Applied to Pollutant Removal on Various Sorbents. *Process Safety and Environmental Protection* **1998**, *76*, 332-340.
